# Supplementary material for: Roles of DJ41_1407 and DJ41_1408 in Acinetobacter baumannii ATCC19606 Virulence and Antibiotic Response
Source: Int J Mol Sci. 2024 Mar 29;25(7):3862. doi: 10.3390/ijms25073862 (PMC11011904; doi:10.3390/ijms25073862)
Supplement: Supplementary file 1 [file ijms-25-03862-s001.zip › ijms-2896251-supplementary.pdf]

Roles of DJ41_1407 and DJ41_1408 in *Acinetobacter baumannii* ATCC19606 aminoglycosides resistance and virulence

Supplementary Material

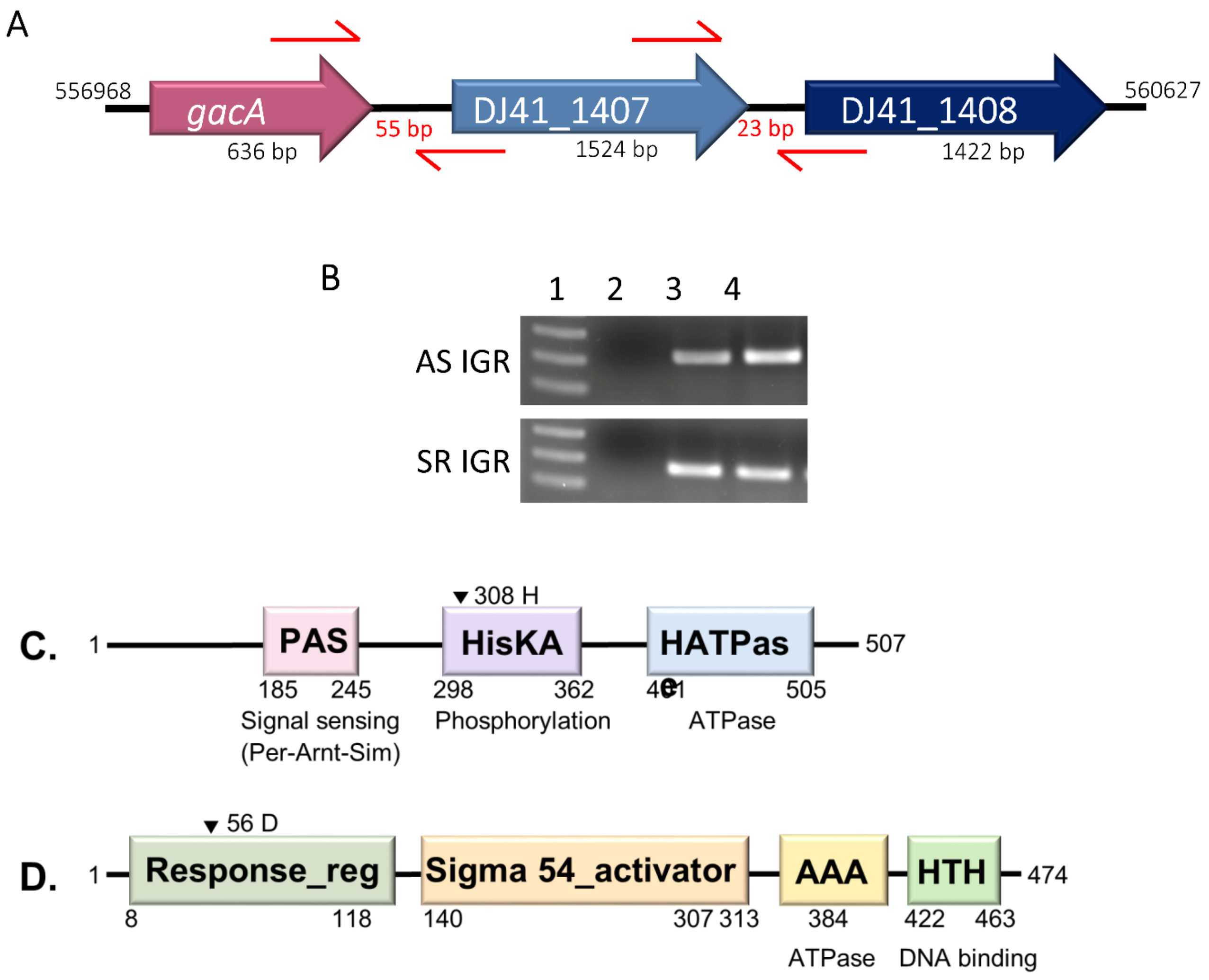

Figure S1. (**A**) Genetic organization of gacA, DJ41_1407 and DJ41_1408 in the *A. baumannii* ATCC19606 genome. gacA (DJ41_1406): 636 bp; DJ41_1407: 1,524 bp; DJ41_1408: 1,422 bp; distance between gacA and DJ41_1407: 55 bp; distance between DJ41_1407 and DJ41_1408: 23 bp; red arrow: position of primers used to amplify the gacA, DJ41_1407 and DJ41_1408 intergenic region. (**B**) Analysis of the PCR products of gacA, DJ41_1407, and the DJ41_1408 intergenic region using 2% agarose gel. 1: 100 bp DNA marker, 2: Negative control, 3: A. baumannii chromosome as template (Positive control), 4: *A. baumannii* cDNA as template. AS IGR: gacA and DJ41_1407 intergenic region amplification. SR IGR: DJ41_1407 and DJ41_1408 intergenic region amplification. Putative domains of DJ41_1407 and DJ41_1408 were analysed using CDvist. (**C**) DJ41_1407 conserved domains. PAS: Per-Arnt-Sim for signal sensing, HisKA: Histidine kinase domain for phosphorylation, HATPase: ATPase domain. The possible phosphorylation site is the histidine residue at 308. (**D**) DJ41_1408 conserved domains. Response_reg: response regulator domain, Sigma 54_activator: domain for transcription initiation, AAA: AAA type ATPase domain, HTH: Helix-turn-helix domain for DNA binding. The possible phosphorylation site is the aspartic acid residue at 56. CDvist links: http://cdvist.zhulinlab.org/

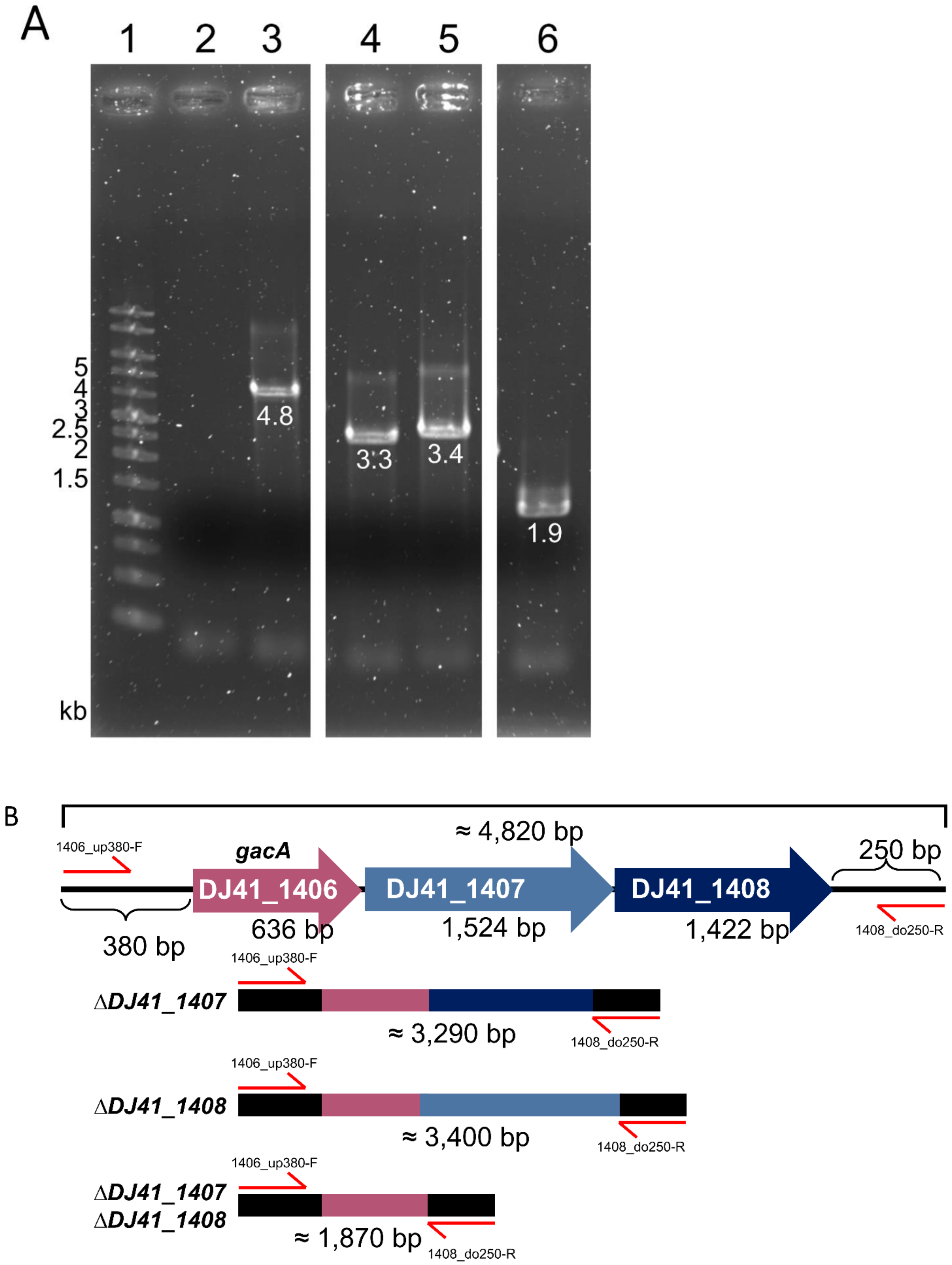

Figure S2: Mutant strain confirmation results. (**A**) Analysis of PCR products of upstream and downstream 250 bp of DJ41_1406-DJ41_1408 operon in all mutants using 0.8% agarose gel. 1: 1 kb DNA marker, 2: Negative control, 3: *A. baumannii* wild-type, 4: ΔDJ41_1407, 5: ΔDJ41_1408, 6: ΔDJ41_1407/08 as template. (**B**) Estimated length for PCR product for each mutant after upstream and downstream 250 bp amplification. Red arrow: primer used in PCR.
